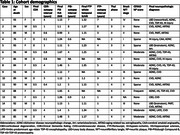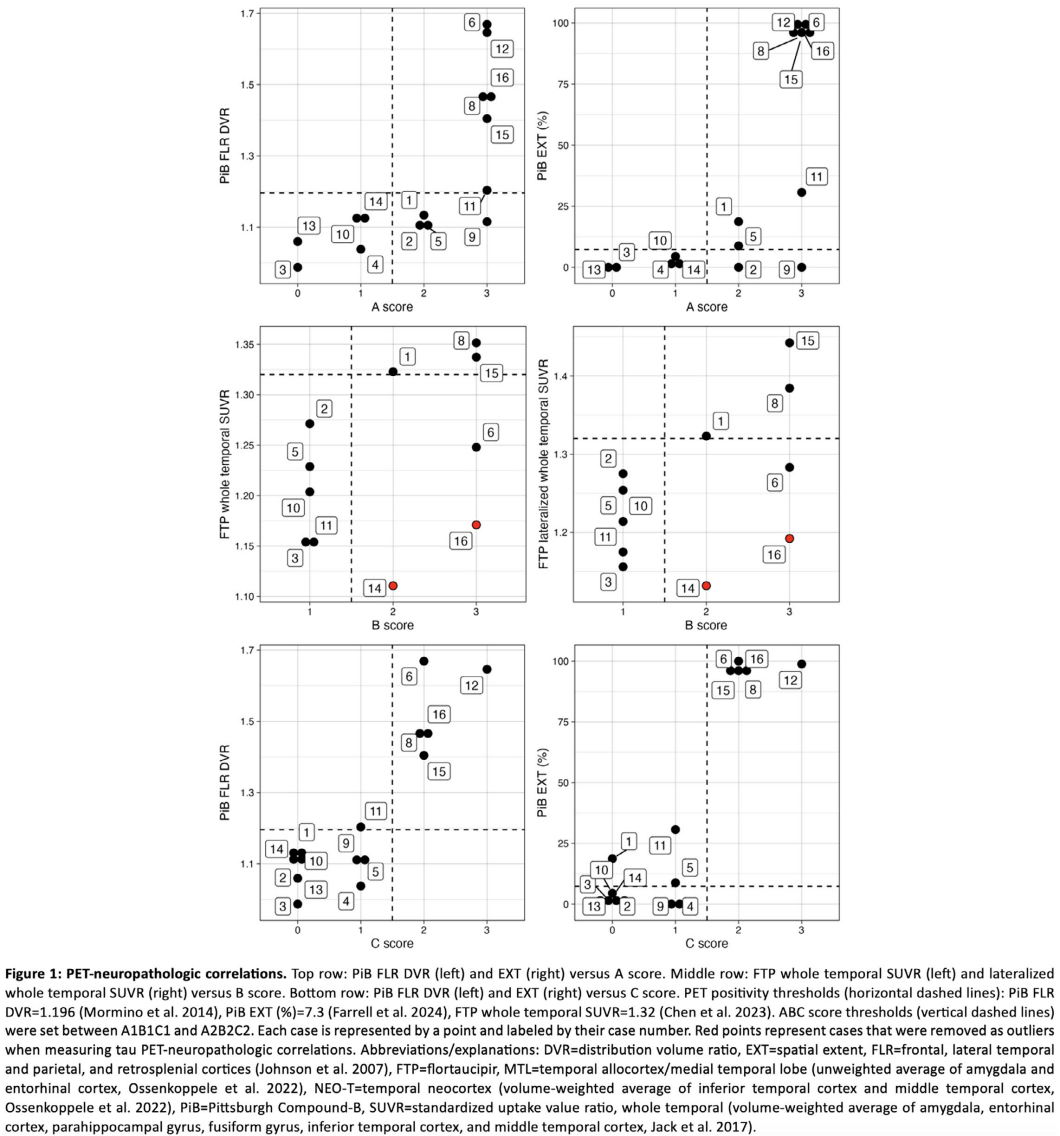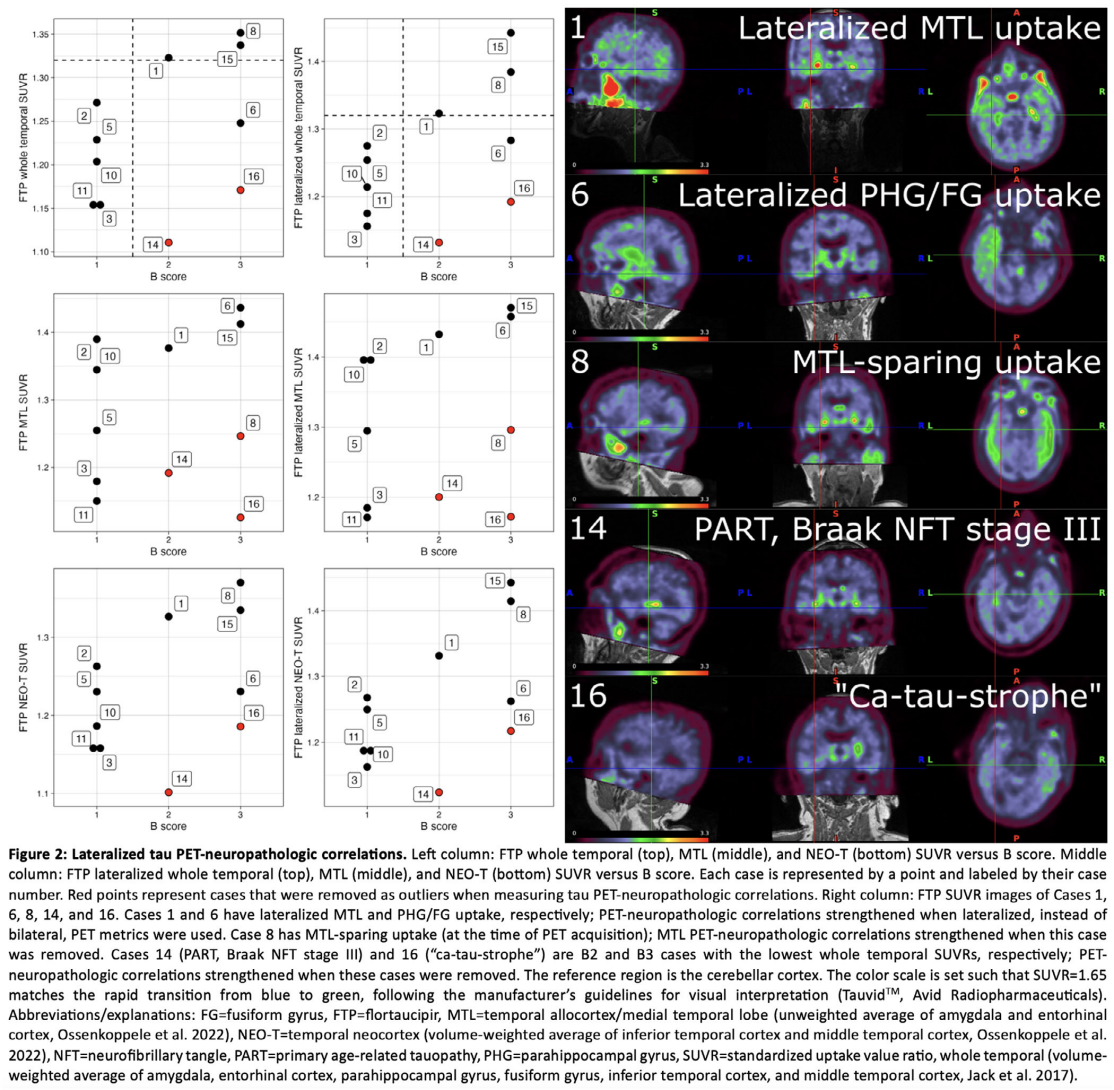# Preliminary autopsy findings from the Harvard Aging Brain Study

**DOI:** 10.1002/alz70862_110188

**Published:** 2025-12-23

**Authors:** Charles D Chen, Cinthya Aguero, Alexandra N. Melloni, Emma G Thibault, Jessie Fanglu Fu, Michelle E. Farrell, Cristina Lois, Teresa Gomez‐Isla, Reisa A. Sperling, Keith A. Johnson, Julie C Price

**Affiliations:** ^1^ Massachusetts General Hospital, Charlestown, MA USA; ^2^ Massachusetts Alzheimer's Disease Research Center, Charlestown, MA USA; ^3^ Gordon Center for Medical Imaging, Massachusetts General Hospital, Boston, MA USA; ^4^ Athinoula A Martinos Center for Biomedical Imaging, Massachusetts General Hospital, Harvard Medical School, Charlestown, MA USA; ^5^ Massachusetts General Hospital, Boston, MA USA; ^6^ Massachusetts General Hospital and Harvard Medical School, Boston, MA USA; ^7^ Center for Alzheimer Research and Treatment, Brigham and Women’s Hospital, Boston, MA USA; ^8^ Massachusetts General Hospital, Harvard Medical School, Boston, MA USA

## Abstract

**Background:**

The Harvard Aging Brain Study (HABS) is a longitudinal observational study on the differences between “normal” aging and preclinical AD. Sixteen HABS participants have undergone brain donation. We evaluate to what extent PET biomarkers predict neuropathologic assessments.

**Method:**

Neuropathologic assessments of amyloid‐β plaques (Thal phase/A score), tau neurofibrillary tangles (Braak NFT stage/B score) and neuritic plaques (CERAD NP score/C score) were compared with antemortem PET. For amyloid‐β PET (PiB), the frontal, lateral temporal, parietal, and retrosplenial cortices (FLR) distribution volume ratio (DVR) and spatial extent (EXT) were calculated. For tau PET (flortaucipir, FTP), the whole temporal, temporal allocortex (MTL), and temporal neocortex (NEO‐T) standardized uptake value ratios (SUVRs) were calculated, using bilateral and lateralized regions (left or right, whichever is greater). PET‐neuropathologic correlations were measured with Spearman’s ρ.

**Result:**

PiB FLR DVR and EXT correlate significantly with A (ρ=0.81, *p* = 0.00029 and ρ=0.73, *p* = 0.0020) and C score (ρ=0.73, *p* = 0.0019 and ρ=0.77, *p* = 0.00087). Two cases had unusually low FTP SUVRs and high B scores (Cases #14 and #16, Table 1). Initially no FTP SUVR correlated significantly with B score. After removing the aforementioned outliers, whole temporal and NEO‐T SUVR correlated significantly with B score (both ρ=0.76, *p* = 0.017, *n* = 9). After additionally removing an MTL‐sparing case (spared at the time of PET acquisition, Case #8), MTL SUVR correlated significantly with B score (ρ=0.78, *p* = 0.021, *n* = 8). Lateralizing SUVRs strengthened correlations with B score for whole temporal (ρ=0.86, *p* = 0.0031, *n* = 9) and MTL (ρ=0.87, *p* = 0.0054, *n* = 8).

**Conclusion:**

Amyloid‐β PET metrics correlated with amyloid‐β plaque spatial distribution and density, while tau PET metrics correlated with tau NFT spatial distribution only after removing outliers. These outliers suggest that PET‐neuropathologic discrepancies arise with tau PET more so than amyloid‐β PET, and were driven by: primary age‐related tauopathy, Braak NFT stage III; amyloid‐β‐driven tau spread (“ca‐tau‐strophe”) between imaging and death; and variability and asymmetry in tau accumulation. In conclusion, individualized tau PET metrics that respect the heterogeneity in tauopathy may be better able to predict neuropathologic assessments.